# Sacroilitis on magnetic resonance imaging in children presenting with low backache: interobserver reliability between radiologists

**DOI:** 10.1186/1546-0096-9-S1-P46

**Published:** 2011-09-14

**Authors:** Athimalaipet Ramanan, Shabina Habibi, M Thyagarajan, M Bradley

**Affiliations:** 1 Bristol Royal Hospital for Children, Bristol, UK; 2 North Bristol NHS Trust, Bristol, UK

## Background

About 25% children with enthesitis related arthritis have sacroilitis at presentation & are at higher risk of developing ankylosing spondylitis. No guidelines exist for the reporting of sacroilitis on MRI in children. Hence there is potentially less agreement between reporting among different radiologists.

## Aims

To assess inter-observer reliability of MRI detection of sacroilitis in children presenting with low backache.

## Methods

Children with low backache underwent MRI of SI joints. T1, T2 & STIR images were obtained in all, & images post-gadolinium contrast obtained in some of the children. Images were reported by two blinded radiologists, with different levels of experience in paediatric musculoskeletal radiology.

## Results

Thirty-one children; 30: images without Gadolinium-contrast; 22: images both with & without Gadolinium-contrast; 1: post-contrast images. The frequency of sacroilitis detected on non-contrast images by radiologist 1 was 32.2% and on post-contrast images was 22.5%, while radiologist 2 detected the same in 29% and 16.1% of the respective images. The overall agreement was moderate (70%) for non-contrast and high (82.6%) for post-contrast images. Mean kappa values were fair (k=0.3077) for non-contrast and moderate (k=0.5534) for post-contrast images. In the 22 children who had both non and post-contrast images, agreement was again moderate for non-contrast (63.64%) and high for post-contrast (81.82%) images. Mean kappa values were slight (k=0.1619) and moderate (k=0.5464) respectively.

## Conclusion

MRI showed fair-moderate level of agreement between radiologists with different levels of experience for the detection of sacroilitis. Images obtained without Gadolinium contrast may overestimate presence of sacroilitis. Standardization and formulation of reporting guidelines will potentially lead to improved reproducibility.

